# LRRK2 Affects Vesicle Trafficking, Neurotransmitter Extracellular Level and Membrane Receptor Localization

**DOI:** 10.1371/journal.pone.0077198

**Published:** 2013-10-22

**Authors:** Rossana Migheli, Maria Grazia Del Giudice, Ylenia Spissu, Giovanna Sanna, Yulan Xiong, Ted M. Dawson, Valina L. Dawson, Manuela Galioto, Gaia Rocchitta, Alice Biosa, Pier Andrea Serra, Maria Teresa Carri, Claudia Crosio, Ciro Iaccarino

**Affiliations:** 1 Department of Clinical and Experimental Medicine, University of Sassari, Sassari, Italy; 2 Department of Biomedical Sciences, University of Sassari, Sassari, Italy; 3 Neuroregeneration and Stem Cell Programs, Institute for Cell Engineering, Johns Hopkins University School of Medicine, Baltimore, Maryland, United States of America; 4 Department of Neurology, Johns Hopkins University School of Medicine, Baltimore, Maryland, United States of America; 5 Department of Physiology, Johns Hopkins University School of Medicine, Baltimore, Maryland, United States of America; 6 Solomon H. Snyder Department of Neuroscience, Johns Hopkins University School of Medicine, Baltimore, Maryland, United States of America; 7 Fondazione Santa Lucia, IRCCS, Rome, Italy; 8 Department of Biology, University of Rome “Tor Vergata”, Rome, Italy; UCL Institute of Neurology, United Kingdom

## Abstract

The leucine-rich repeat kinase 2 (*LRRK2*) gene was found to play a role in the pathogenesis of both familial and sporadic Parkinson’s disease (PD). LRRK2 encodes a large multi-domain protein that is expressed in different tissues. To date, the physiological and pathological functions of LRRK2 are not clearly defined. In this study we have explored the role of LRRK2 in controlling vesicle trafficking in different cellular or animal models and using various readouts. In neuronal cells, the presence of LRRK2^G2019S^ pathological mutant determines increased extracellular dopamine levels either under basal conditions or upon nicotine stimulation. Moreover, mutant LRRK2 affects the levels of dopamine receptor D1 on the membrane surface in neuronal cells or animal models. Ultrastructural analysis of PC12-derived cells expressing mutant LRRK2^G2019S^ shows an altered intracellular vesicle distribution. Taken together, our results point to the key role of LRRK2 to control vesicle trafficking in neuronal cells.

## Introduction

Most Parkinson’s disease (PD) cases occur sporadically and several genes associated with monogenetic forms of the disease have been identified in patients [1]. Mutations in the leucine-rich repeat kinase 2 gene (LRRK2, PARK8) cause late-onset, autosomal dominant PD that is clinically and neurochemically indistinguishable from idiopathic forms [2], [3]. The LRRK2 gene encodes a large protein of 2527 amino acids belonging to the ROCO protein family [4]. Similar to other ROCO proteins, LRRK2 contains a Ras-of-complex (Roc) GTPase domain and a C-terminal of Roc (COR) domain in conjunction with a protein kinase domain with close homology to members of the mixed-lineage and receptor-interacting protein kinase families. LRRK2 also contains a number of repeat domains (armadillo, ankyrin, leucine-rich repeats and C-terminal WD40 repeats that surround the central Roc-COR-kinase catalytic region) of uncertain function. Interestingly, multiple amino acid substitutions of the same residue R1441 (R1441C, R1441G, and R1441H) in the highly conserved GTPase domain and multiple mutations (I2012T, G2019S, and I2020T) in the kinase domain have been identified in patients [5]. The most common pathological mutation G2019S increases the kinase activity of LRRK2 by 2–3 fold [6], [7], however some other mutations show an unchanged or reduced kinase activity [8]. Studies from several independent groups have evaluated the frequency of LRRK2 mutations in many different populations, and such mutations have been found not only in 3–5% of familial PD but also in approximately 1–3% of idiopathic PD cases [9]. Despite extensive studies based on both animal and cellular models, the pathological role of LRRK2 in PD onset and progression is still largely unclear, and LRRK2 substrate(s) remain fairly elusive. To date, LRRK2 has been involved in different physiological functions ranging from miRNA processing [10] to translation regulation [11], cytoskeleton organization [12], [13], [14], autophagy-lysosomal pathways [15], [16] and immunoregulation [17]. Different experimental approaches seem to suggest a potential role of LRRK2 in vesicle trafficking [18]. Firstly, LRRK2 appears to be localized in different intracellular compartments that play a critical role in the control of vesicular trafficking: endoplasmic reticulum, Golgi apparatus and associated vesicles, cytoskeleton, lipid rafts and synaptic vesicles [19], [20]. Secondly, alteration in dopamine (DA) release has been described in different LRRK2 transgenic rodents, ranging from a reduction in DA extracellular content without [14], [21] or with pharmacological manipulation [21], [22] to an increased DA extracellular content in transgenic WT LRRK2 mice [22] or in transgenic rats expressing mutant LRRK2^G2019S^ [23]. In primary neuronal cells, LRRK2 silencing perturbs vesicle dynamics and distribution within the recycling pool, leading to a significant decrease in docked vesicles but an increase in the amount of vesicle recycling [24]. Moreover, alteration of LRRK2 expression by knockdown of endogenous LRRK2 in primary neuronal cells significantly impairs synaptic vesicle endocytosis [24], [25], but a similar effect was observed following LRRK2 overexpression [25] thus leaving unclear which is the exact role of this protein.

In this work we have analysed the role of LRRK2 in controlling neurotransmitter extracellular levels as well as the neurotransmitter receptor membrane levels through different experimental approaches. Taken together, our results point to a key role of LRRK2 in controlling vesicle trafficking and distribution.

## Materials and Methods

### Animals

Mice were housed and treated in strict accordance with the NIH Guide for the Care and Use of Laboratory Animals. All animal procedures were approved by the Institutional Animal Care and Use Committees of the Johns Hopkins Medical Institutions (Animal Welfare Assurance No. A3272-01). Mice were maintained in a pathogen-free facility and exposed to a 12 h light/dark cycle with food and water provided *ad libitum*.

### Reagents and Solutions

Antibodies: anti-TH (1∶4000 Sigma), anti-Myc (1∶5000 Sigma), anti-DRD1 (1∶2000 Sigma), anti-NR1 (1∶2000 Sigma), anti-Sec8 (1∶4000 BD-Biosciences), anti-clathrin (1∶5000 BD-Biosciences). Reagents: Tween® 20 (Polyethylene glycol sorbitan monolaurate), Phenylmethanesulfonyl fluoride (PMSF), protease inhibitor cocktail and (−)-Nicotine hydrogen tartrate salt were obtained from Sigma-Aldrich (Milano, Italy). LRRK2 inhibitor GSK2578215A was from Tocris. The phosphate-buffered saline (PBS) solution was made using NaCl (137 mM), KCl (2.7 mM), Na2HPO4 (8.1 mM), KH_2_PO_4_ (1.47 mM), CaCl_2_ (1.19 mM), MgCl_2_ (0.54 mM), and glucose (7.5 mM) from Sigma and then adjusted to pH 7.4. Dulbecco’s modified Eagle’s medium (DMEM)–F12, Streptomycin/Penicillin, Hygromycine B, Geneticin-G418 were purchased from Invitrogen and doxycycline from BD Biosciences. The tetracycline-free Fetal Bovine Serum (FBS) was from Lonza Sales Ltd (Switzerland).

### Plasmid Constructions

The plasmids for inducible expression or LRRK2 were obtained by digestions of cDNAs corresponding to human LRRK2 (WT or R1441C or G2019S) in fusion with 5X myc repeats [26] with *Bam*HI and *Xho*I and subcloned in *Bam*HI/*Eco*RV cloning sites in pTRE2 vector (Clontech).

cDNA coding for mouse growth hormone (GH, NM_008117.2) was RT–PCR amplified from mouse pituitary gland mRNA (oligo forward: ATCAGGATCCTTGGCAATGGCTACAGACTC, reverse: ATCAGGATCCGAAGGCACAGCTGCTTTCC), digested with *Bam*HI restriction enzyme and cloned in *Bam*HI cloning site of pCS2-5X-myc-tag containing the tag in C-terminal position. Plasmid pTL2-DRD1 (kindly provided by E. Borrelli, University of California, Irvine) was used as template for DRD1 cDNA, the PCR fragment (oligo forward: ATCCTCGAGAAGATGGCTCCTAACACTTCTACCA, reverse: CTCCTCGAGGGTTGAATGCTGTCCGCTGTG) was digested with *Xh*oI and subcloned into pcDNA3.1-3X-flag-tag containing the tag in the C-terminal position. p-TK-Hyg (Clontech Laboratories Inc ) was used to impart hygromycin resistance to PC12 ON cells.

### Cell Lines and PC12 Stable Clones

Human neuroblastoma SH-SY5Y cells (ATCC number CRL-2266) were grown in DMEM-F12 (Invitrogen), 10% fetal calf serum (FCS, Invitrogen) at 37°C. The PC12-TET-ON cell line (Clontech Laboratories Inc) was cultivated in DMEM-F12 supplemented with 10% Tetracycline-free FCS (Lonza) at 37°C.

The plasmid pTRE2 vectors containing cDNAs coding for LRRK2 variants (WT or R1441C or G2019S) were co-transfected with p-TK-Hyg in a 8∶1 molar ratio into PC12-TET-ON cells, using Lipofectamine® LTX Reagent (Life Technologies) according to the manufacturer’s protocol. The different PC12-TET-ON clones were maintained under selection by 400 µg/mL of G418 and 200 µg/mL of hygromicin-B. Individual clones expressing both antibiotic resistances were picked after 14 days of selection, moved in a 96 well plate, and maintained in selective medium till confluence growth. Different individual clones were analyzed for LRRK2 expression upon induction by doxycycline (0.2 µg/mL).

### Analysis of Intracellular and Extracellular Dopamine and Metabolites

Intracellular and extracellular dopamine (DA), 3-methoxytyramine (3-MT), 3,4-Dihydroxyphenylacetic acid (DOPAC), and homovanillic acid (HVA) were determined by HPLC with electrochemical detection as previously described [27]. In brief, cells were lysed in 250 µL 1% metaphosphoric acid containing 1 mM EDTA. After centrifugation (17,500 g for 10 min at 4°C), the supernatant was filtered, and a 15- µL aliquot was immediately injected into the HPLC system.

In each experiment, 100×10^3^ cells/cm^2^ were plated and treated 24 h later (time 0) with doxycycline 0.2 µg/mL. After 48 h, the medium was aspirated from each well and stored, and the cells were collected in metaphosphoric acid. Samples were subsequently analyzed for levels of total DA (DA+3-MT) and its metabolites DOPAC and HVA in cell lysates and incubation medium. Values in cell lysate were expressed as nanomoles per milligram of protein. Total cell extract protein concentration was determined using the method of Lowry et al. (1951).

### Capillary Tube Construction for *in vitro* Microdialysis

The capillary tube for microdialysis of PC12 cell lines is an adaptation of an *in vitro* device described previously [28], [29]. The microdialysis probe was constructed using two sections of plastic-coated silica tubing (150 µm o.d., 75 µm i.d., Scientific Glass Engineering, Milton Keynes, UK), each placed in the centre of a semipermeable polyacrylonitrile dialysis fiber (AN-69, Hospal Industrie, Meyzieu, France). Each semipermeable membrane had an active length of 40 mm. Then each section of plastic-coated silica tubing was positioned in the centre of polyethylene tubing (0.58 mm i.d., 35 mm long, Portex). This section of silica tubing served as the inlet. Dialysates from polyacrylonitrile dialysis fiber were collected from polyethylene tubing, which served as the outlet. All parts were coated with quick-drying epoxy glue. Afterwards the microdialysis probe (the semipermeable polyacrylonitrile dialysis fiber plus sealed plastic-coated silica tubing) was placed in non heparinized microhematocrit capillary tubes (7.5 mm long, 1.1 mm i.d., Chase Scientific Glass, Rockwood, IL, USA). The final volume of microdialysis chamber was approximately 50 µL.

### Microdialysis Procedures

Microdialysis experiments were performed during the exponential phase of cell growth. 5×10^4^ cells/cm^2^ were plated and treated 24 h later (time 0) with different Doxycycline concentrations. After 48 h cells were washed twice using 5 ml of modified PBS and 10% DMEM (perfusion medium), harvested and centrifuged (94 g for 5 min). Cells were resuspended in PBS/DMEM and the number of cells/ml was assessed in a Burker chamber. The initial volume of the cell suspension was eventually adjusted to reach a final concentration of 1×10^6^ cells/50 µL. Nicotine (5 mM) effect on DA secretion from PC12 lines was evaluated by means of microdialysis *in vitro* as previously described [30].

The cellular microdialysis probe was perfused with PBS/DMEM by means of a peristaltic microinfusion double-channel pump (P720 peristaltic pump (Instech, Plymouth Meeting, PA, USA), which pumped PBS/DMEM at a flow rate of 3.0 µL/min. The pump channels were connected to the inlet by a length of polythene tubing. The perfusion apparatus was then filled with 50 µL of the PC12 cell suspension by aspiration, which was performed manually by means of a 1.0 mL syringe connected to the plastic coated silica tubing sealed outside the polythene tubing. Thereafter, the perfusion apparatus was kept at 37°C. After 1 h of stabilization, 3 microdialysis samples (60 µL each) were recovered at 20 min intervals. Nicotine was added to the perfusion medium and removed after 60 min. In case of LRRK2 inhibitor treatments, GSK2578215A (1 µM) was added at the beginning of stabilization. Samples were recovered during the next two hours. Subsequently, a 35 µL aliquot of each collected dialysate was analyzed by HPLC. The concentration of neurochemicals detected after the first 20 min of perfusion was taken as time 0 concentration. Cell viability was assessed before the start and at the end of each experiment by trypan blue exclusion. The viability rate was given as the difference between final and initial percentage of non-viable cells [29], [30].

### Chromatographic Analysis of Dialysates from PC12 Cell Suspension

DA was quantified in dialysates of selected experiments (1.0×10^6^ cells) by HPLC–EC, as described previously [29] using an Alltech 426 HPLC pump (Alltech, Sedriano, Italy) equipped with a Rheodyne injector (model 7725, Rohnert Park, CA, USA), a column (15 cm, 4.6 mm i.d., ODS80TM C18, Toso Haas, Stuttgart, Germany), an electrochemical detector ANTEC–Leyden EC controller (ANTEC, Zoeterwoude, The Netherlands), and a PC-based ADC system (Varian Star Chromatographic Workstation, Varian, Walnut Creek, CA, USA). The mobile phase was citric acid (0.1 M), ethylenediaminetetraacetic acid (EDTA, 1.0 mM), methanol (8.7%) and sodium octylsulfate (48 mg/L), with a flow rate of 1.2 mL/min and pH 2.9.

### Transient Transfections and Analysis of GH Secretion

Transient expression of each vector was performed with Lipofectamine LTX Reagent (Life Technologies) according to the manufacturer’s instructions. After an incubation of 4–6 h with transfection reagents, the cells were cultured in normal growth medium for 24 or 48 h. For GH secretion analysis, SH-SY5Y cells (1.0×10^5^ cells) were seeded in 24 mm plates and co-transfected the following day either with GH-5Xmyc and pCS2-MTK empty vector or with GH-5Xmyc and the different pCS2-5Xmyc-LRRK2 isoforms in a ratio of 1∶10. 24 hours after transfection, the cells were washed twice with fresh medium and normal growth medium was added for another 16 h. In case of LRRK2 inhibitor treatments, GSK2578215A was added 1 h before medium change and then after medium change. The extracellular medium was then collected and centrifuged at 10000×g for 10 min to eliminate cell debris, while the cells were washed twice with PBS and immediately lysed by Laemmli buffer 1X. For DRD1 membrane localization experiments, the cells were co-transfected in 6 cm plates as described above (in a ratio of 1∶5 respectively for DRD1-3Xflag and 5Xmyc-LRRK2 or empty vector) for 48 hours. The quantification of either GH-5Xmyc or DRD1-3Xflag in the different fractions was performed by western blot analysis and densitometric evaluation of the obtained bands (Quantity-One Biorad).

### Subcellular Fractionation of Cells or Mouse Tissues

Tissues from 2 months old LRRK2^WT^ or LRRK2^G2019S^ transgenic mice [16] were quickly dissected and frozen. Subcellular fractionation was conducted as described in [31]. Briefly, SH-SY5Y or HEK293 cells or striatum were homogenized in ice-cold homogenization-buffer (320 mM sucrose, 4 mM HEPES, pH 7.4, protease inhibitor cocktail from Sigma). The homogenates were centrifuged at 1000×g for 10 min to produce the pellet containing nuclei and large debris fraction (P1). The supernatant (S1) was further fractionated into pellet (P2 containing the membrane fraction) and supernatant (S2) by centrifugation at 10,000×g for 20 min. The S2 was ultracentrifuged at 100,000×g to obtain the pellet (P3 containing the vesicle fraction). Protein content was determined using the Bradford protein assay. Equal amount of protein extracts were loaded into the SDS-PAGE.

### Western Blot Analysis

Western blot analysis was performed as previously described [32]. Briefly, protein content was determined using the Bradford protein assay. Equal amount of protein extracts were resolved by standard SDS/PAGE. Samples were electroblotted onto Protan nitrocellulose (Schleicher & Schuell GmbH). Membranes were incubated with 3% low-fat milk in 1×PBS-Tween 0.05% solution with the indicated antibody for 16 h at 4°C. Anti-Rabbit IgG (whole molecule)- and Anti-Mouse IgG (whole molecule)-Peroxidase antibody were used to reveal immunocomplexes by enhanced chemioluminescence (Pierce).

### Statistical Analysis

Concentrations of neurochemicals in dialysates from PC12 cell suspension were expressed in µM and given as mean ± SEM. Drug effects on neurochemicals were statistically evaluated in terms of changes in absolute dialysate concentrations. Differences within or between groups were determined by paired or unpaired t-tests (ANOVA followed by Student–Newman–Keuls post-hoc analysis). The null hypothesis was rejected when p<0.05.

## Results

### Generation and Characterization of PC12 Cells Stably Expressing Doxycycline-inducible WT or Pathological Mutant LRRK2s

**Figure 1:**
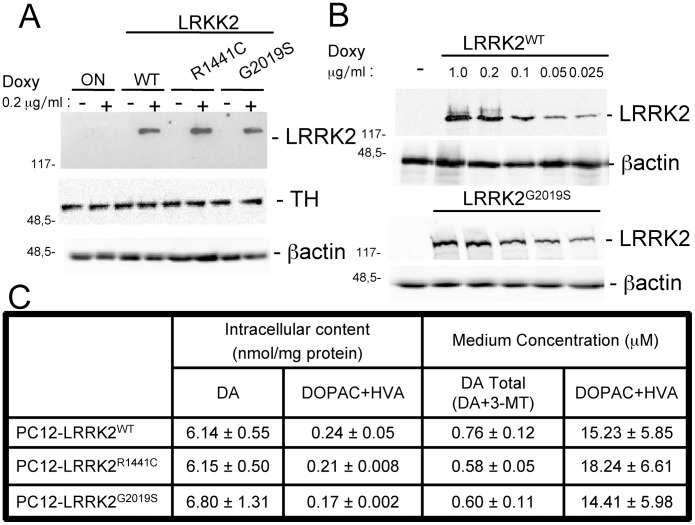
Characterization of PC12 cell expressing WT or mutant LRRK2. (A) PC12-derived cells expressing myc-tagged human WT or mutant-LRRK2 were left untreated (−) or treated (+) for 48 h with 0.2 µg/mL doxycycline to induce expression of transgenic LRRK2. Cell lysates were subjected to reducing SDS/PAGE. The anti-myc antibody was used to visualize LRRK2 and anti-TH for tyrosine hydroxylase. β-actin serves as controls for equal loading of samples. (B) Dose-dependent expression of doxycyline-inducible LRRK2^G2019S^. Cells were treated for 48 h with the indicated concentration of doxycycline and equal amounts of protein were tested in Western blot analysis with anti-myc or anti-β-actin antibodies. (C) Effect of doxycycline on DA and DOPAC+HVA concentration in PC12 cell lysates and extracellular medium after 48 h expression. At the beginning of each experiment, 10^5^ PC12 cells/cm^2^ were plated and after the desired incubation period, the medium was aspirated from each well and stored, and the cells were collected in metaphosphoric acid. Samples were subsequently analyzed for levels of DA and its metabolites DOPAC and HVA in cell lysates and incubation medium. Results are the means ± SEM of three experiments performed in triplicate.

Plasmid constructs for wild type LRRK2 (LRRK2^wt^) or for pathological mutants LRRK2^G2019S^ and LRRK2^R1441C^ were transfected in PC12-ON cell lines. After several weeks of selection by hygromicin, single stable clones were isolated. Different clones were characterized and finally three clones expressing comparable level of LRRK2 protein after 48 h of induction by 0.2 µg/mL doxycycline treatment were chosen (Figure 1A upper panel). All cell lines expressed comparable levels of Tyrosine Hydroxylase (TH) after the same treatments (Figure 1A, middle panel). A higher dose of doxycycline (1 µg/mL) does not further increase the LRRK2 expression level (Figure 1B). 48 hours after doxycycline treatment, we analysed the intracellular and extracellular dopamine (DA) level and its metabolism products in the different cell lines (Figure 1C). All the stable clones show comparable intracellular and extracellular levels of DA content as well as the intracellular and extracellular level of DOPAC+HVA, allowing the comparative analysis between the different stable clones performed in the following experiments.

### LRRK2 Influences the Basal and Nicotine-induced Secretion of DA in PC12 Cells

**Figure 2:**
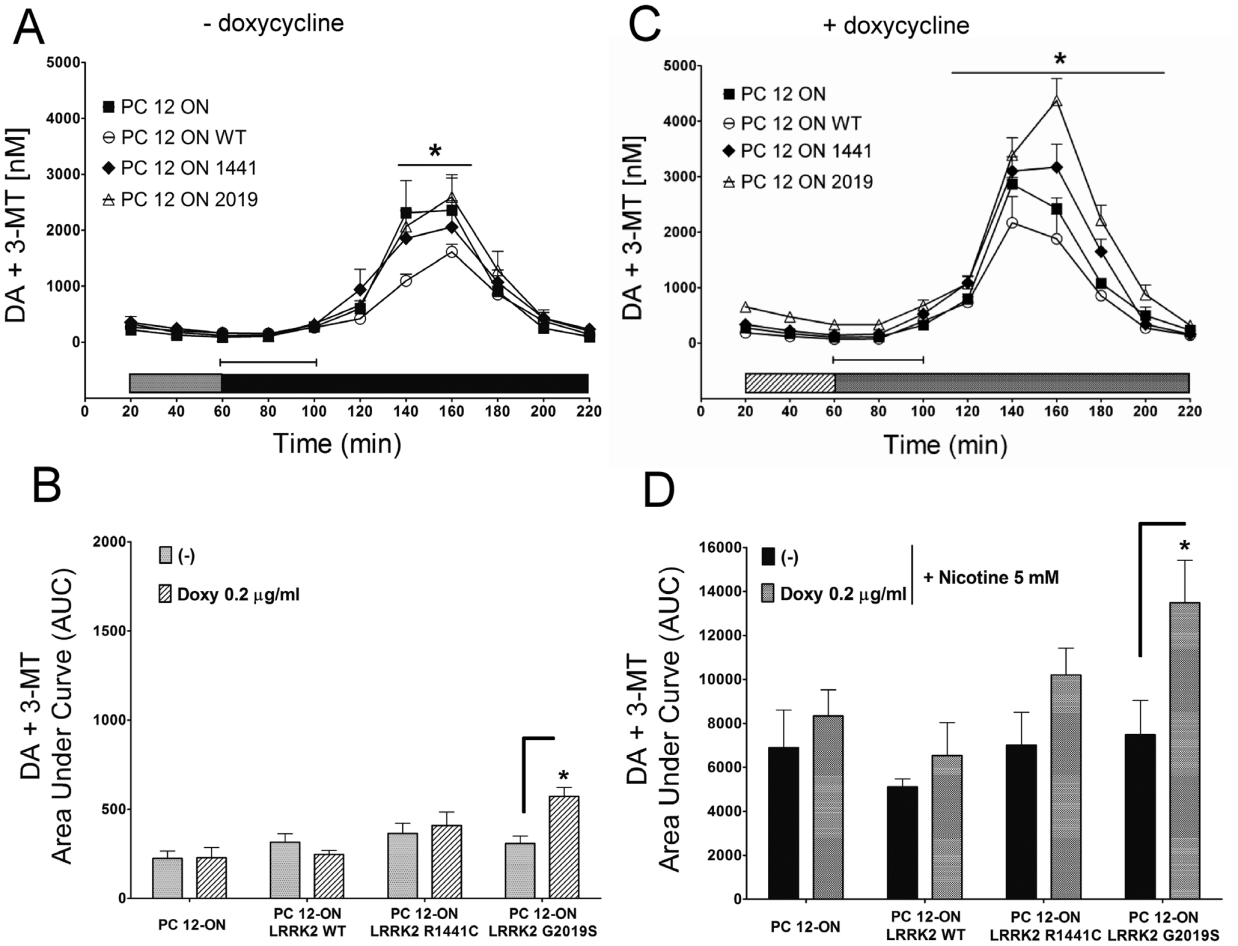
Effect of LRRK2 expression on dopamine (DA+3-MT) extracellular level. PC12-derived cell lines were left untreated (A) or treated (C) for 48 h with 0.2 µg/mL doxycycline. After 60 minutes of stabilization, three baseline dialysates were collected at 20-minute intervals [gray bars in panel (A), and gray bars with stripes in panel (C)]. Starting from 60 minutes, nicotine was infused for 60 minutes (capped lines). Microdialysates were continuously recovered during drug infusion and after nicotine discontinuation [black bars in panel (A), and dotted bars in panel (C)]. Values are mean ± SEM and refer to dopamine concentrations in dialysates. Statistical significance was assessed using analysis of variance (ANOVA) for differences over time determined by Newman-Keules *t* test and unpaired *t*-tests. *p<0.05 compared with pertinent baseline values of all groups. Graphs in panel (B) and (D) represent the area under curve (AUC) values. (B) Basal dopamine concentration in dialysates of PC12 cell lines untreated (gray bars corresponding to the same color in panel A) or treated with 0.2 µg/mL doxycycline [gray bars with stripes corresponding to same color in panel (C)]. (D) Dopamine concentrations in dialysates integrated after nicotine administration in PC12 cell lines untreated [black bars corresponding to same color in panel (A)] or treated with 0.2 µg/mL of doxycycline [dotted bars corresponding to same color in panel (C)]. AUC values are mean ± SEM. *p<0.05 vs corresponding control.

In order to evaluate the effect of the expression of LRRK2 on basal and nicotine-induced secretion of DA, we performed a microdialysis study on cells [33] treated with 0.2 µg/mL doxycycline for 48 h compared with untreated (−) cells. This doxycycline dose was chosen because, in preliminary experiments, it did not show any significant effect on DA secretion on PC12-ON cells in contrast to 1 µg/mL of doxycycline that had a negative effect on DA secretion (data not shown). Microdialysates were collected at 20 minute intervals after 1 h of stabilization (Figure 2A–B–C–D). The time course analysis of basal and nicotine induced dopamine release in the absence (Figure 2A) or presence (Figure 2C) of doxycycline is shown. In Figure 2B–D, the Area Under Curve (AUC) of the different samples is shown; in particular, the baseline of DA+3-MT is represented from the AUC of points 20–60 minutes before nicotine treatment in absence (Figure 2B bars) or presence of doxycycline (Figure 2B barred bars), while the DA+3-MT nicotine-induced release is represented by the AUC related to points 80–220 minutes in absence (Figure 2D black bars) or presence of doxycycline (Figure 2D dotted bars). The expression of LRRK2^G2019S^ induces an 85% increase (p<0.05) vs corresponding control in the basal level of total extracellular DA (DA+3-MT), as shown in figure 2C (20–40–60 minutes) compared to 2A (20–40–60 minutes) and quantified by the AUC in Figure 2B. In contrast, under the same experimental conditions expression of either LRRK2^WT^ or LRRK2^R1441C^ does not induce a significant increase in total DA extracellular levels (Figure 2A–C and quantified in 2B). Interestingly, the presence of mutant LRRK2 determines an increase in nicotine-induced DA compared to PC12-ON or LRRK2^WT^ cells (Figure 2C 60–220 minutes *vs* 2A 60–220 minutes). Quantification by AUC of secreted DA indicates an increase of 22% (although not statistical significant) and 61% (p<0.05), respectively for LRRK2^R1441C^ and LRRK2^G2019^ (Figure 2D).

**Figure 3:**
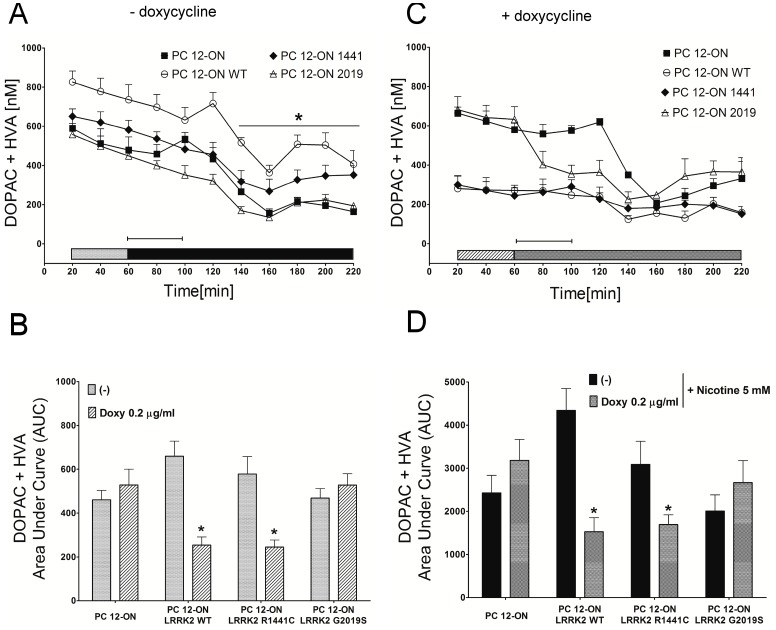
Effect LRRK2 expression on DOPAC+HVA concentrations. PC12-derived cell lines were left untreated (A) or treated (C) for 48 h with 0.2 µg/ml doxycycline. After 60 minutes of stabilization, three baseline dialysates were collected at 20-minute intervals [bars in panel (A), and gray bars with stripes in panel (C)]. Starting from 60 minutes, nicotine was infused for 60 minutes (capped lines). Microdialysates were continuously recovered during drug infusion and after nicotine discontinuation [black bars in panel (A), and dotted bars in panel (C)]. Values are mean ± SEM and refer to DOPAC+HVA concentrations in dialysates. Statistical significance was assessed using analysis of variance (ANOVA) for differences over time determined by Newman-Keules *t* test and unpaired *t*-tests. *p<0.05 compared with pertinent baseline values of all groups. Graphs in panel (B) and (D) represent the area under curve (AUC) values. (B) Basal DOPAC+HVA concentration in dialysates of PC12 cell lines untreated [bars corresponding to the same color in panel (A)] or treated with 0.2 µg/mL doxycycline [bars with stripes corresponding to the same color in panel (C)]. (D) DOPAC+HVA concentrations in dialysates integrated after nicotine administration in PC12 cell lines untreated [black bars corresponding to the same color in panel (A)] or treated with 0.2 µg/ml doxycycline [dotted bars corresponding to the same color in panel (C)]. AUC values are mean ± SEM. *p<0.05 vs corresponding control.

Under the same experimental conditions, we measured extracellular levels of DOPAC+HVA. A significant change both in the basal condition and after nicotine infusion is observed following doxycycline treatment in PC12 cells expressing LRRK2^WT^ or LRRK2^R1441C^ (Figure 3A–B–C–D). In particular the values are −61.4% for WT and −51.7% for LRRK2^R1441C^ in doxycycline treated samples and −64.9% for WT and −45.3% for R1441C mutant by doxycycline and nicotine treatment. No significant differences were observed in PC12-ON cells or expressing the LRRK2^G2019S^ mutant.

**Figure 4:**
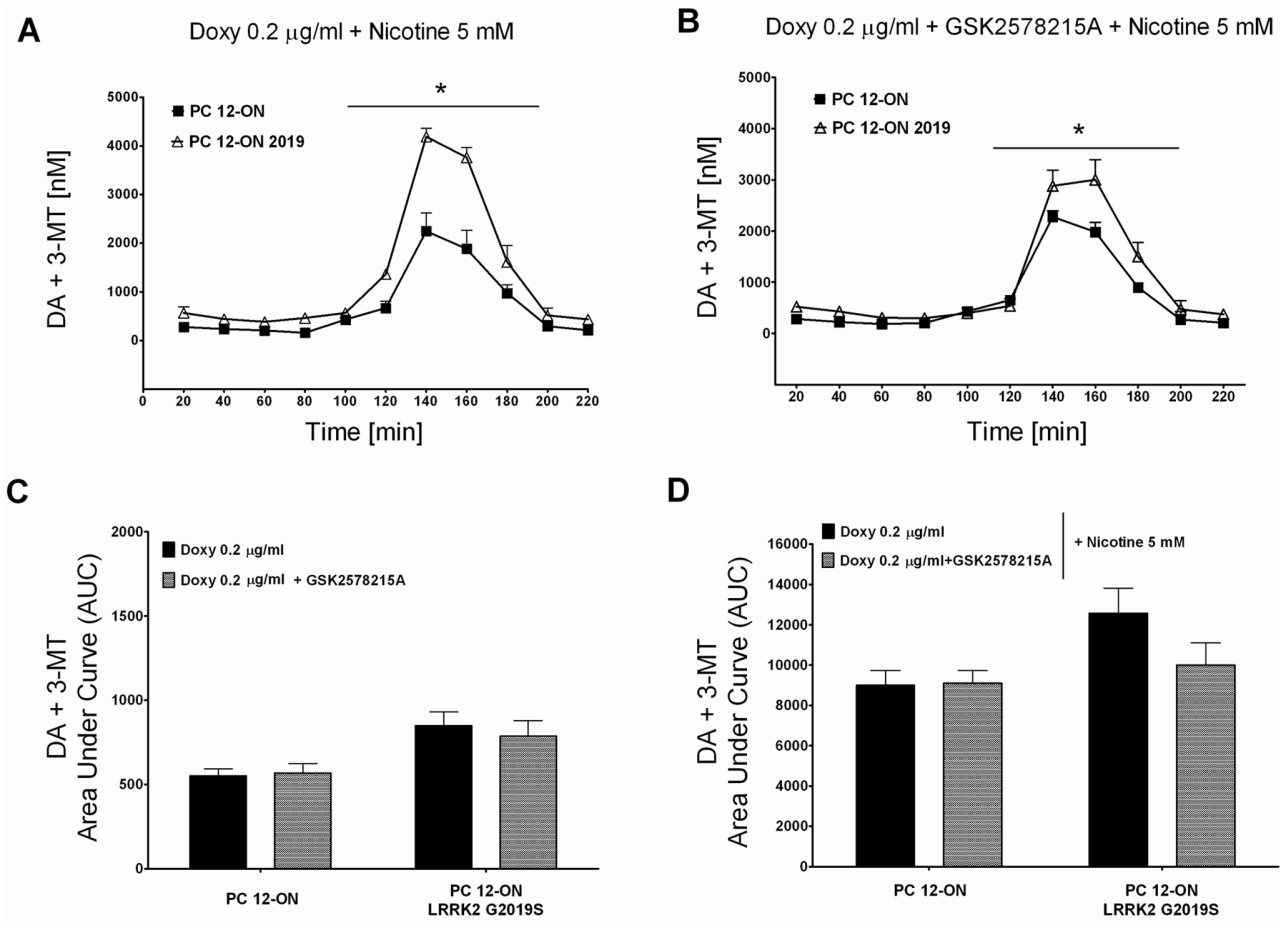
Effect of LRRK2 inhibitor GSK2578215A on dopamine (DA+3-MT) extracellular level. PC12-derived cell lines were left untreated (A) or treated (B) by 1 µM of GSK2578215A. After 60 minutes of stabilization, three baseline dialysates were collected at 20-minute intervals as previously described. Starting from 60 minutes, nicotine was infused for 60 minutes. Values are mean ± SEM and refer to dopamine concentrations in dialysates. Statistical significance was assessed using analysis of variance (ANOVA) for differences over time determined by Newman-Keules *t* test and unpaired *t*-tests. *p<0.05 compared with pertinent baseline values of all groups before nicotine treatment. Graphs in panel (C) and (D) represent the area under curve (AUC) values. (C) Basal dopamine concentration in dialysates of PC12 cell lines untreated or treated with 1 µM of GSK2578215A (minutes 20–40–60 before nicotine treatment of Figure 4A *vs* B). (D) Dopamine concentrations in dialysates integrated after nicotine administration in PC12 cell lines untreated or treated with 1 µM of GSK2578215A (from minutes 80 to 220 after nicotine treatment of Figure 4A *vs* B). AUC values are mean ± SEM.

To evaluate the importance of LRRK2 kinase activity on increased DA extracellular level, we used the LRRK2 inhibitor GSK2578215A on PC12-ON or PC12-ON expressing the LRRK2^G2019S^. The presence of LRRK2 inhibitor does not influence the DA extracellular level in basal conditions (20–40–60 minutes in figure 4A *vs* B or the relative AUC in Figure 4C) in both cellular types. In presence of nicotine, the inhibitor does not affect the expected increase in DA extracellular level in PC12-ON cells while it determines a reduction in PC12-ON expressing the LRRK2^G2019S^ mutant although this DA extracellular level decrease does not rich statistical significance in three independent experiments (Figure 4A from 60 to 220 minutes *vs* 4B from 60 to 220 minutes and the relative AUC in Figure 4D).

Under the same experimental conditions, the GSK2578215A inhibitor does not affect the concentration of DOPAC+HVA (Figure S1).

### Vesicle Distribution in PC12 Cells Expressing LRRK2^G2019S^ Pathological Mutant

**Figure 5:**
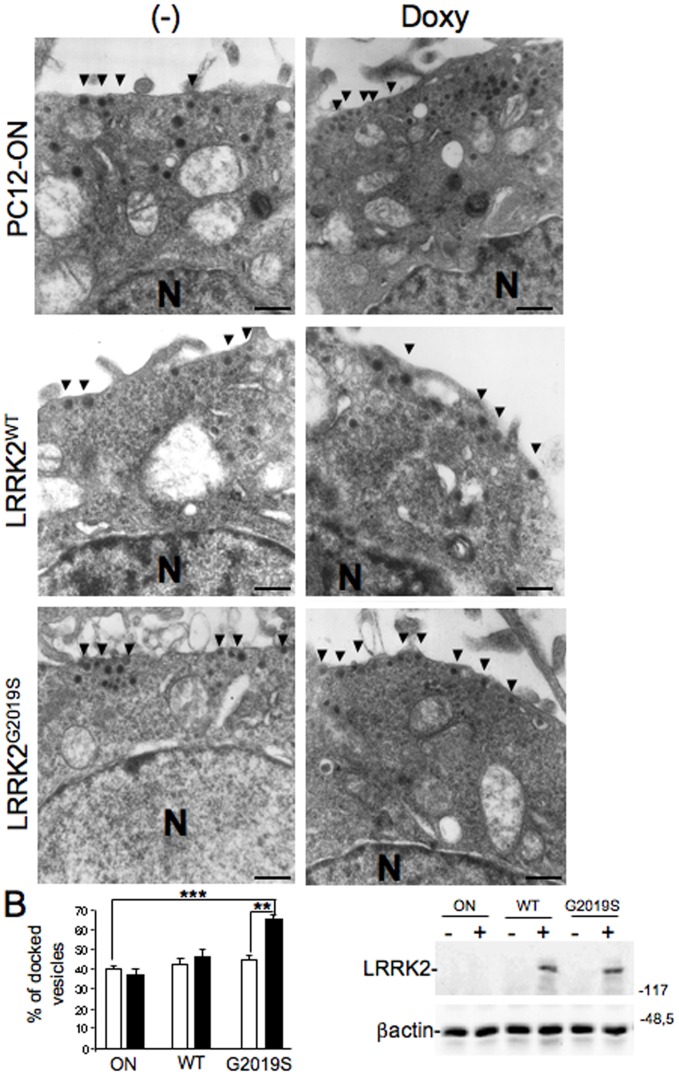
Effect of LRRK2^G2019S^ expression on vesicle distribution. (A) Transmission electron micrographs of PC12-ON or PC12-LRRK2^WT^ or PC12-LRRK2^G2019S^ cells untreated (−) or treated with 0.2 µg/ml doxycycline for 48 h (+). Arrows point at vesicles near the membrane. N indicates the nucleus. Scale bars = 0,5 µM (B) Quantification of data in (A). Bars represent the mean ± SEM (n = 30 cells/sample). **p<0.01 comparing cells expressing or not LRRK2^G2019S^ as indicated. (C) Control Western blot analysis using anti-myc (to detect LRRK2) and anti-βactin (as loading standard) on cells treated as in (A).

Given the dopamine secretion changes associated with LRRK2 expression, we asked whether LRRK2 might be involved in synaptic vesicle (SV) movement/distribution. Using an electron microscopy approach we analysed the vesicles distribution in PC12-ON or PC12-ON-LRRK2^G2019S^ cell lines. Expression was induced for 24 hours with 0.2 µg/mL doxycycline and cells were quickly fixed for electron microscopy. Non-induced cells served as a control. Analysis of SV dimensions or relative abundance did not show significant differences between the different groups (data not shown). Conversely LRRK2 expression seemed to influence synaptic vesicle distribution in terms of shorter distance to the plasma membrane. We observed a clear increase in the relative abundance of vesicles located in close proximity to the membrane in presence of LRRK2^G2019S^ mutant compared to the same cells not expressing the mutant or to PC12 ON cells either treated or untreated with doxycycline (Figure 5A–B). The level of LRRK2 expression in the different cell lines was analysed by Western blot using an aliquot of cells before fixation and it is shown in Figure 5C.

### LRRK2 Increases Growth Hormone Extracellular Level in Neuronal Cells

**Figure 6:**
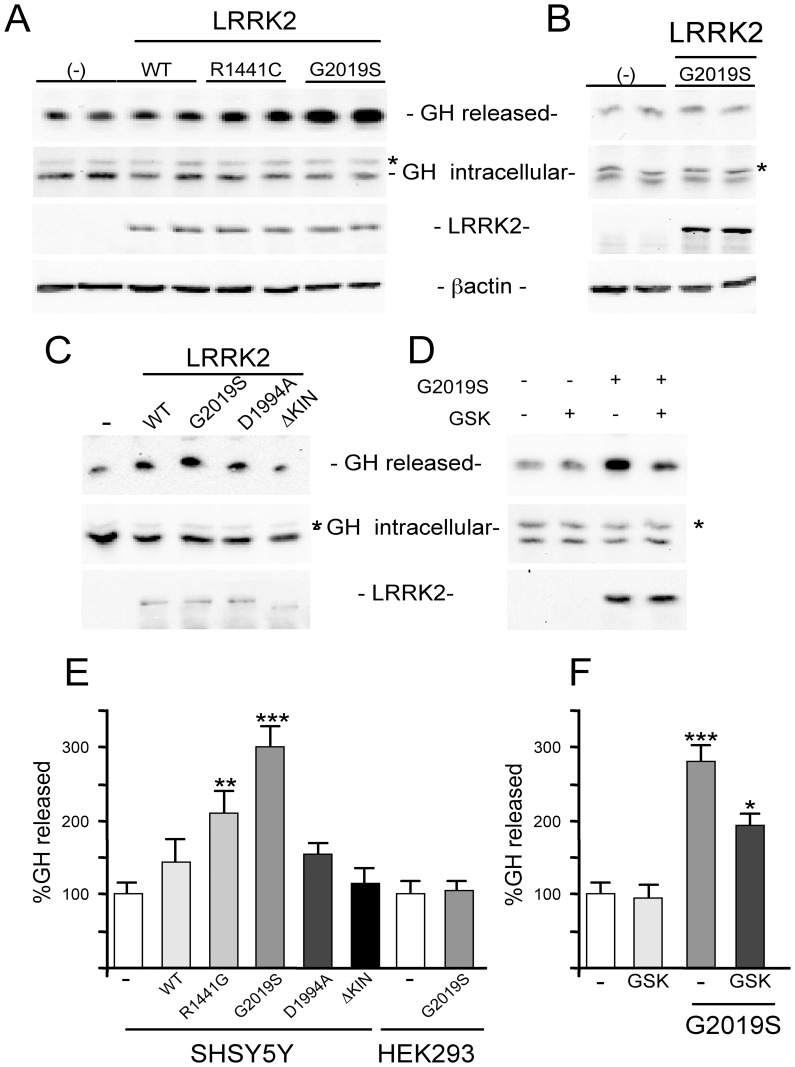
Effect of LRRK2 expression on released GH from neuronal and non-neuronal cells. (A) SH-SY5Y were transfected with plasmids coding for GH alone (−) or with the indicated mutant LRRK2s. Western blot on the extracellular medium (released GH) or cell protein extracts were performed using anti-myc antibodies for GH or LRRK2 and anti-β-actin as controls for equal loading of samples. The asterisk (*) indicates a non-specific band. (B) HEK293 cells were transfected with GH alone (−) or with LRRK2^G2019S^. Western blot on the extracellular medium (released GH) or cell protein extracts were performed using Anti-myc antibody for GH or LRRK2 and anti-β-actin as controls for equal loading of samples. The asterisk (*) indicates a non-specific band. (C) Analysis of LRRK2^D1994A^ or LRRK2^Δkinase^ on GH extracellular level in the same experimental conditions. (D) Effect of LRRK2 inhibitor on GH extracellular level in the same experimental conditions (E) Quantification by Student’s *t* test of data obtained in (A–B–C). **p<0,01 and ***p<0,001 versus (−). (F) Quantification by Student’s *t* test of data obtained in (D). *p<0,05 and ***p<0,001 versus (−).

To further evaluate the involvement of LRRK2 in the mechanisms of vesicle trafficking we generated a plasmid construct to follow secreted proteins that consists of mouse growth hormone (mGH), containing a myc-tag at the C-terminal position. GH secretion has been largely used to follow neuronal secretion, since GH is localized predominantly in vesicles in regulated secretory pathways [34], [35]. We co-transfected mGH alone or together with different LRRK2 constructs in both neuronal (SH-SY5Y or PC12) or non-neuronal (HEK293) cells. 24 h after co-transfections the cell medium was changed and the level of GH secretion was evaluated collecting the extracellular medium 16 h later. As shown in figure 6A, the presence of LRRK2^WT^ in SH-SY5Y determines a slight increase in extracellular GH level. This level is further increased in the presence of mutant LRRK2, with LRRK2^G2019S^ inducing the maximum effect (Figure 6A–E). Total cell lysates were used to analyse the intracellular level of mGH, LRRK2 and β-actin (Figure 6A). Similar results were obtained in PC12 cells although these cells had been transfected with lower efficiency compared to SH-SY5Y (data not shown). Interestingly, the effect of LRRK2 on GH secretion was not visible performing similar experiments in kidney HEK293 cells (Figure 6B–E) suggesting a possible neuronal-specific mechanism of action for LRRK2.

In order to understand the role of LRRK2 kinase activity in the control of GH extracellular level we decided to use two different approaches. First we analysed the effect of two LRRK2 dead kinase mutants carrying a deletion (delta-kinase) or point mutation (D1994A). Surprisingly, the absence of kinase domain completely abolishes the LRRK2 effect on GH extracellular level, while LRRK2^D1994A^ has an effect roughly comparable to LRRK^WT^ (Figure 6C). To further evaluate the importance of LRRK2 kinase activity we used the LRRK2 inhibitor GSK2578215A. The presence of LRRK2 inhibitor significantly reduces the increase in GH extracellular level due to LRRK2^G2019S^ expression (Figure 6D–F).

### LRRK2 Affects the Localization of Dopamine Receptor D1 both in Neuronal Cells and Transgenic Mouse Tissues

**Figure 7:**
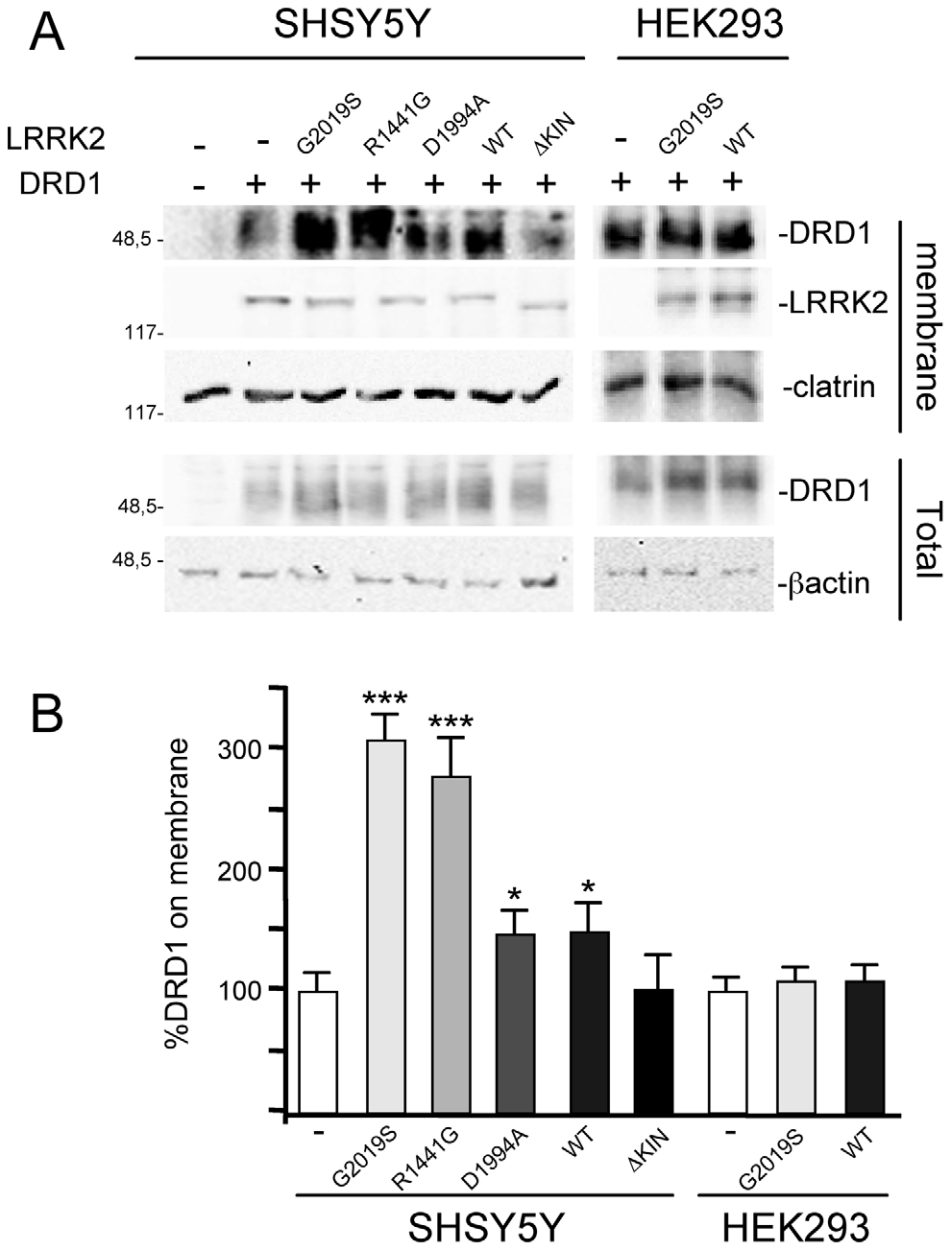
Effect of LRRK2 on Dopamine D1 receptor (DRD1) distribution on neuronal and non-neuronal cells. A) SH-SY5Y or HEK293 were transfected with DRD1 alone (−) or with the indicated mutant LRRK2. Western blot on the total and membrane fraction proteins were performed using anti-flag antibody for DRD1 or anti-myc for LRRK2 and anti-β-actin or clathrin as controls for equal loading of samples. Note that for SDS-PAGE analysis the protein samples were not boiled as suggested in anti-DRD1 data sheet. (B) Quantification by Student’s *t* test of data obtained in (A). *p<0,05 **p<0,01 and ***p<0,001.

**Figure 8:**
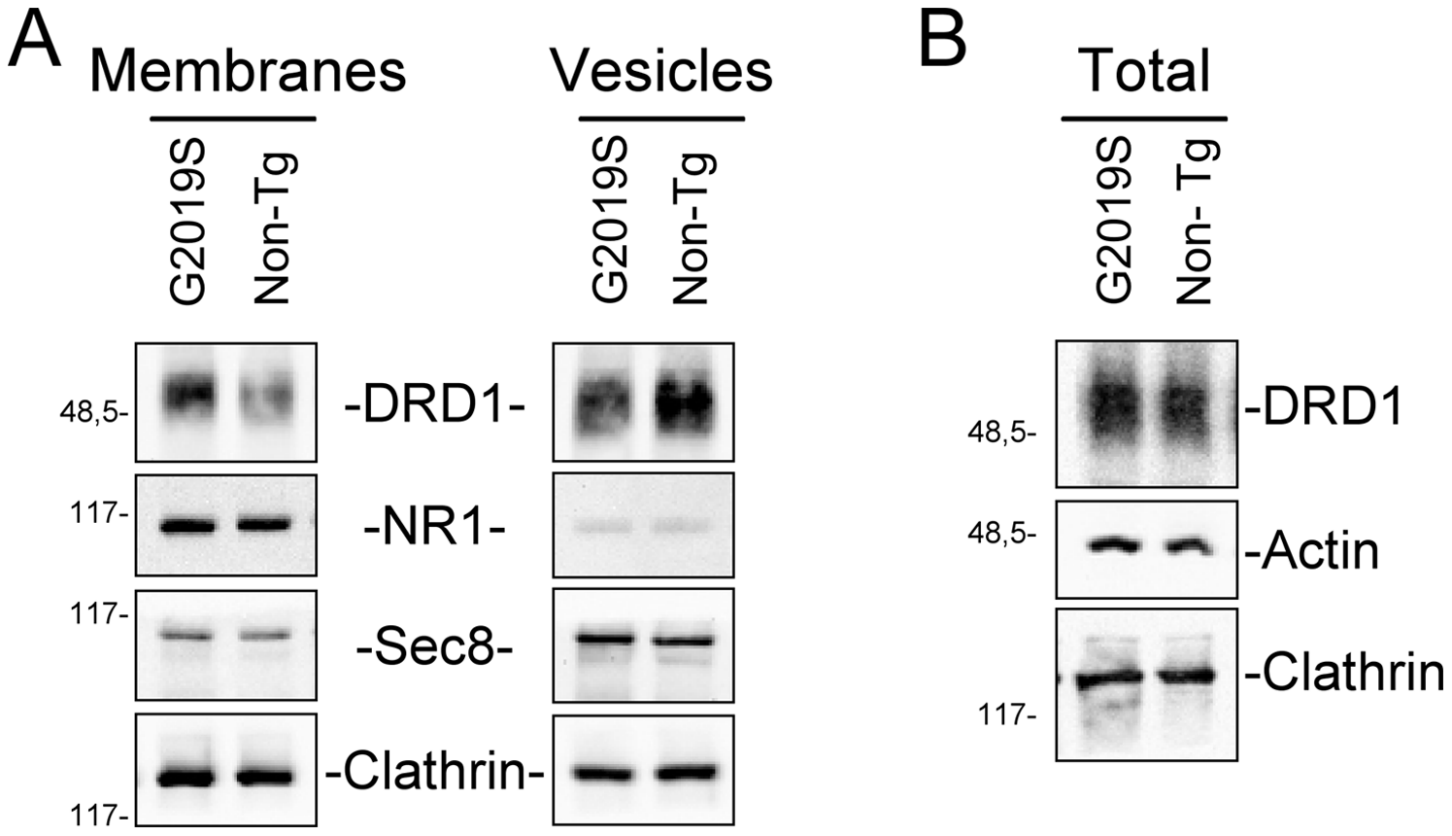
Effect of LRRK2 on Dopamine D1 receptor (DRD1) distribution on the striatum of G2019S LRRK2 or non-transgenic mice. The total, membrane or vesicle fractions were purified from 5 different LRRK2^G2019S^ transgenic mice or relative control (No Tg). Each of the five fractions were quantified and pooled together. A) Analysis of DRD1 level on protein extracts from membrane or vesicle fraction obtained from striatum of No Tg or LRRK2^G2019S^ transgenic mice. The western blot was performed using anti-DRD1, anti-NR1, anti-Sec8, or anti-clathrin antibodies. Note that for DRD1 analysis the samples were not boiled for the SDS-PAGE analysis as recommended in the antibody data sheet. B) Analysis of DRD1 level on total protein extracts obtained from striatum of No Tg or LRRK2^G2019S^ transgenic mice. The Western blot was performed using anti-DRD1, anti-actin or anti-clathrin antibodies. Note that for DRD1 analysis the samples were not boiled for the SDS-PAGE analysis.

Vesicle trafficking is a complex process regulating multiple different cellular functions, including neurotransmitter or protein release and localization of membrane receptors. The previous results prompted us to analyse the possible effect of LRRK2 on membrane receptor localization using the membrane levels of dopamine receptor D1 (DRD1) as a read-out. DRD1is highly expressed in the prefrontal cortex, striatum and nucleus accumbens, although it is absent in dopaminergic cells where Dopamine D2 receptors are expressed [36]. We co-transfected SH-SY5Y cells with plasmid coding for DRD1 in absence or in presence of one among LRKK2^WT^, LRKK2^G2019S^, LRKK2^R1441G^, LRKK2^D1994A^ or LRKK2^Δkinase^. 48 hours after transfection, the sub-cellular distribution of DRD1 receptors was analysed by cell fractionation. As shown in figure 7, the presence of LRRK2^G2019S^ and LRKK2^R1441G^, and, to a lesser extent, of LRRK2^WT^ or LRRK2^D1994A^ determines a significant increase in the level of membrane-associated DRD1 compared to controls with DRD1 alone, while the effect is completely abolished in presence of LRKK2^Δkinase^. In agreement with previous findings on GH secretion, this effect is not observed in HEK293 cells (Figure 7B, D). To extend our analysis to *in vivo* models we analysed the distribution of two neurotransmitter receptors in the striatum of transgenic LRRK2^G2019S^ mice compared to non-transgenic mice. In particular, we analysed the NMDA-NR1 receptor (NR1) and DRD1 distribution in total, membrane or vesicle fractions obtained from the striatum of 5 different animals of the two genotypes. The samples were independently prepared, quantified and pooled together before SDS-PAGE loading using the same amount of protein. As shown in Figure 8A the expression of LRRK2^G2019S^ leads to a significant increase of DRD1 in membrane fraction paralleling a significant decrease in vesicle fraction, with no significant differences in total protein extracts between the two genotypes (Figure 8B). No significant differences in the different fractions were observed for NR1, clathrin or sec8, a member of the exocyst complex.

## Discussion

In neurons, vesicle trafficking mediates neurotransmitter or protein release and uptake, localization of membrane receptors, changes in plasma membrane composition at the cell surface and, not least, organelle biogenesis, thus underlying virtually all functions of the nervous system. The transport, binding and fusion of secretory vesicles with the cell surface are tightly regulated and their dysregulation has been implicated in PD and other neurological disorders [37]. There are two exocytosis pathways, constitutive and regulated, by which secretory proteins are released from cells. Regulated exocytosis is triggered by signals originating outside the cell, usually through a rise in Ca^2+^ concentration at release sites; however, the regulated pathway also exhibits basal or unstimulated secretion, which is distinct from constitutive secretion [38]. Moreover, there is now a general consensus that synaptic vesicles formation relies predominantly on the endocytic pathway [39] strongly suggesting a prominent role of endocytosis in the control of neurotransmitter extracellular level or membrane receptor level. Neurons and neuroendocrine cells (for example, PC12 cells) have both constitutive and regulated exocytosis, while most non-neuronal cells have only constitutive secretion. Chromaffin PC12 cells have been extensively used as cellular model of dopaminergic neurons to study vesicle trafficking under basal or stimulated conditions [40]. In fact, PC12 cells mimic many features of central DA neurons; they are characterized by the presence of catecholamine synthesizing enzymes (i.e. tyrosine hydroxylase) [41], catecholamine storage granules of about 0.2–0.5 µm [41], [42] and DA receptors, as well as by DA uptake mechanisms. Like dopaminergic neurons, PC12 cells also contain the type A isoform of monoamine oxidase (MAO) [43]. Finally, we and other researchers have demonstrated that PC12 cells release catecholamines in response to different stimulations [28], [30], [44]. Moreover, PC12 cells together with SH-SY5Y neuronal cells have been largely used to analyse the LRRK2 physio-pathological functions [6], [26], [45], [46].

In this study, we highlight an important cellular function of LRRK2 in the regulation of vesicle trafficking. We demonstrate that, under different experimental conditions, the presence of PD-linked mutant LRRK2 determines an increase in DA extracellular level in short-time dynamic experiments in PC12 cells, an increase in GH release in SH-SY5Y cells and an increase of DRD1 on cell membrane of both SH-SY5Y cells and striatum of LRRK2^G2019S^ transgenic mice. The content of intracellular DA, DOPAC and HVA and DA, 3-MT, DOPAC and HVA in culture medium is similar in all the LRRK2-containing clones, whether or not expression of the protein has been induced (Figure 1C). This result suggests that LRRK2 expression does not interfere with basal dopamine metabolism. An alternative explanation is that long time sampling of extracellular content (48 h) may mask small/significant differences in DA secretion and metabolism among different PC12 clones since catecholamine medium content is the result of several biological phenomena including secretion, reuptake and enzymatic oxidative metabolism, but as well as the outcome of chemical events such as autoxidation of hydroxyl groups [28], [30]. However, short-time dynamic experiments performed using *in vitro* microdialysis demonstrate a basal increase of DA secretion in cells expressing LRRK2^G2019S^ and a nicotine-induced increase of extracellular DA in those expressing both mutated LRRK2 genes (Figure 2A–B). Interestingly, these results on PC12 cells match that of LRRK2^G2019S^ transgenic rat model [23], in which temporally induced, but not constitutive, over-expression of LRRK2^G2019S^ determines an increased DA extracellular level and an increased motor activity, probably due to an impairment in dopamine reuptake by dopamine transporter [23]. In our PC12 cells, LRRK2 expression negatively affects the DA enzymatic metabolism, resulting in a decrease of DOPAC+HVA in basal microdialysate samples with the exception of the LRRK2^G2019S^ cells and the same trend is emphasized by nicotine treatment. This difference may be explained by the increased DA secretion in LRRK2^G2019S^-expressing cells with the consequent reuptake and rise of the substrate available for intracellular MAO-A enzymes.

The expression of LRRK2^G2019S^ also affects the vesicle distribution in PC12 cells in terms of shortest distance from cell membranes. Although our ultrastructural analysis does not discriminate univocally the three different SV classes [47], it has generally been assumed that the vesicles closest to release sites (in a range of 100 nm) and the docked vesicles represent the readily releasable pool (RRP) and thus are recruited first during synapse activity [48]. Thus, our electron microscopy results may suggest that the increase in extracellular DA level either in basal conditions or after nicotine stimulation may be due to alteration in vesicle distribution inside the cells.

The increase in DA level in the extracellular medium of PC12 expressing LRRK2^G2019S^ is comparable to the biological effect detectable in SH-SY5Y cells using GH or DRD1 as read-out in the presence of LRRK2 pathological mutants (Figure 6–7). To date, the role of kinase activity in LRRK2 physio-pathological function is still controversial because different pathological or risk factor mutants exist with unchanged or reduced kinase activity (for review see Greggio et al. 2009) and because in different experimental models the kinase dead LRRK2 mutants behave as the pathological mutants [49]. Under our experimental conditions, the LRRK2^D1994A^ mutant protein shows smaller but consistent effect on both DRD1 membrane level and secreted GH compared to pathological mutants despite the fact that LRRK2^D1994A^ has a robust reduction in kinase activity [6], [26]. In agreement with this result, the LRRK2 inhibitor GSK2578215A reduces but does not fully eliminate the LRRK2^G2019S^ effect either on DA or GH extracellular level. Taken together, our results seem to suggest that LRRK2 may primarily act as a regulatory scaffolding protein, where the LRRK2 kinase activity may further support this function. The absence of biological effect of LRRK2 lacking the kinase domain (LRRK2^ΔKIN^) on both GH and DRD1 readouts may be in part explained by a destabilization of the protein due to the large amino acid deletion.

Interestingly, the LRRK2 biological effects, in our experimental conditions, seem to be neuronal specific, since no increase in GH extracellular level or DRD1 membrane level was observed in HEK293 cell when mutant LRRK2 is present. Neuronal-specific mechanisms of vesicle trafficking are well described [38] and this aspect could explain at least partially the neuron specific toxicity of mutant LRRK2. This view is supported by experiments *in vivo*, showing that the LRRK2 effects on DRD1 localization in neuronal cells is observed in the striatum of G2019S transgenic mice. The transgene expression determines an increase of DRD1 in the plasma membrane fraction that correlates with a decrease in the vesicle fraction. This effect is possibly specific for this receptor since it is not detectable for NMDA-NR1 receptor. It is generally accepted that membrane receptors show different turnover rate and different endocytic pathways. For instance, NMDARs are considered to be relatively stable once delivered to the cell surface of mature neurons, especially when compared with AMPARs that, even under basal conditions, cycle much more rapidly to and from the surface [50]. On the other hand, DRD1s internalize rapidly following agonist-induced activation [51]. Thus, the LRRK2 protein does not seem to exert a general control in secretion but rather accurately modulates the vesicle turnover.

The amount of secreted neurotransmitter or plasma membrane protein at steady state level is the sum of the transport reaching the cell surface from biosynthetic pathways, the leaving of the surface via the endocytic pathway, and the returning to plasma membrane from the intracellular endosomal pools; consequently, a change in any of these parameters could be responsible for the differences that we observe in DA or GH release and DRD1 membrane localization. For instance, LRRK2 has been implicated to different extents in vesicle trafficking either directly throughout endocytosis regulation [24], [25], [49] or indirectly throughout interaction with actin or tubulin [12], [52] that in turn may affect vesicle movement among the cytoskeleton. The dysregulation on vesicle trafficking may have important consequences on neuronal toxicity. It is believed that inter-related processes like oxidative stress, excitotoxicity, inflammation, mitochondrial dysfunction and altered proteolysis are involved in the cascade of events that will lead to DA neuron death [53]. In this respect, the increase in neurotransmitter levels in the inter-synaptic space or the increase in membrane receptors in neuronal plasma membrane due to the expression of mutant LRRK2 could alter the normal neuronal physiology that in turn may lead to neuronal toxicity. In this respect, the increased neurotransmitter levels in the inter-synaptic space or the increase in membrane receptors in neuronal plasma membrane due to the expression of mutant LRRK2 could alter the normal neuronal physiology that in turn may lead to neuronal toxicity. For instance, a dysregulation in DA level has been extensively studied as prominent PD etiological factor for the neurotoxic properties of DA metabolites [54], [55].

## Supporting Information

Figure S1 **Effect of LRRK2 inhibitor GSK2578215A on DOPAC+HVA concentrations.** PC12-derived cell lines were left untreated (A) or treated (B) with 1 µM of GSK2578215A. After 60 minutes of stabilization, three baseline dialysates were collected at 20-minute intervals as previously described. Starting from 60 minutes, nicotine was infused for 60 minutes. Microdialysates were continuously recovered during drug infusion and after nicotine discontinuation. Values are mean ± SEM and refer to DOPAC+HVA concentrations in dialysates. Statistical significance was assessed using analysis of variance (ANOVA) for differences over time determined by Newman-Keules *t* test and unpaired *t*-tests. *p<0.05 compared with pertinent baseline values of all groups before nicotine treatment. Graphs in panel (C) and (D) represent the area under curve (AUC) values. (C) Basal DOPAC+HVA concentrations in dialysates of PC12 cell lines untreated or treated with 1 µM of GSK2578215A (minutes 20–40–60 before nicotine treatment of Figure S1A *vs* B). (D) DOPAC+HVA in dialysates integrated after nicotine administration in PC12 cell lines untreated or treated with 1 µM of GSK2578215A (from minutes 80 to 220 after nicotine treatment of Figure S1A *vs* B). AUC values are mean ± SEM.
(TIF)
