# Supplementary figures and images for: LRRK2 Affects Vesicle Trafficking, Neurotransmitter Extracellular Level and Membrane Receptor Localization
Source: PLoS One. 2013 Oct 22;8(10):e77198. doi: 10.1371/journal.pone.0077198 (PMC3805556; doi:10.1371/journal.pone.0077198)

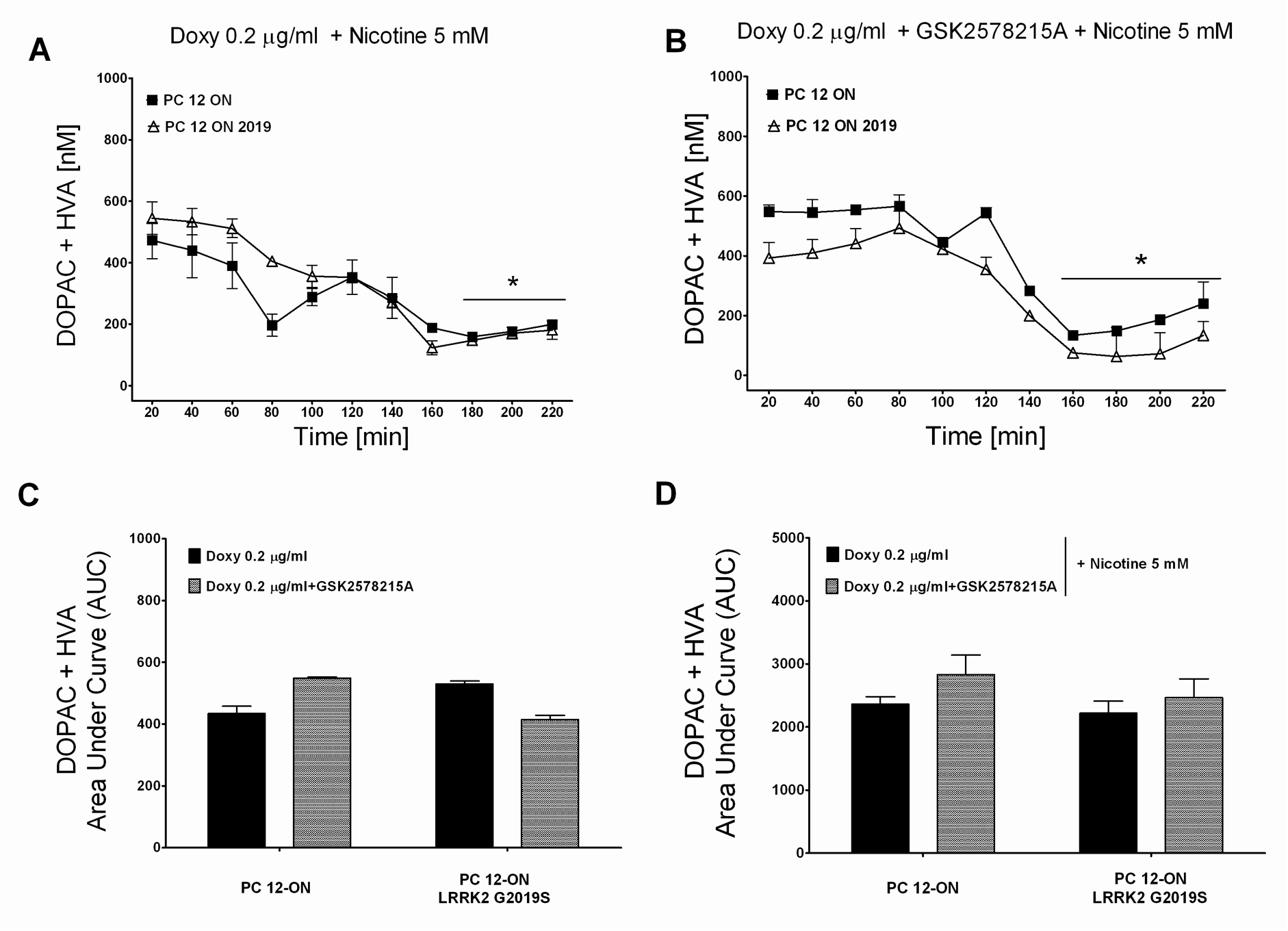

Supplement: Figure S1 — Effect of LRRK2 inhibitor GSK2578215A on DOPAC+HVA concentrations. PC12-derived cell lines were left untreated (A) or treated (B) with 1 µM of GSK2578215A. After 60 minutes of stabilization, three baseline dialysates were collected at 20-minute intervals as previously described. Starting from 60 minutes, nicotine was infused for 60 minutes. Microdialysates were continuously recovered during drug infusion and after nicotine discontinuation. Values are mean ± SEM and refer to DOPAC+HVA concentrations in dialysates. Statistical significance was assessed using analysis of variance (ANOVA) for differences over time determined by Newman-Keules t test and unpaired t-tests. *p<0.05 compared with pertinent baseline values of all groups before nicotine treatment. Graphs in panel (C) and (D) represent the area under curve (AUC) values. (C) Basal DOPAC+HVA concentrations in dialysates of PC12 cell lines untreated or treated with 1 µM of GSK2578215A (minutes 20–40–60 before nicotine treatment of Figure S1A vs B). (D) DOPAC+HVA in dialysates integrated after nicotine administration in PC12 cell lines untreated or treated with 1 µM of GSK2578215A (from minutes 80 to 220 after nicotine treatment of Figure S1A vs B). AUC values are mean ± SEM. (TIF) [file pone.0077198.s001.tif]
